# A review of key microbial and nutritional elements for mechanistic modeling of rumen fermentation in cattle under methane-inhibition

**DOI:** 10.3389/fmicb.2024.1488370

**Published:** 2024-11-21

**Authors:** Eleanor M. Pressman, Ermias Kebreab

**Affiliations:** Department of Animal Science, University of California, Davis, Davis, CA, United States

**Keywords:** methanogenesis, mathematical model, differential equation, 3-nitrooxypropanol, bromoform

## Abstract

The environmental impacts of livestock agriculture include the production of greenhouse gasses (GHG) such as methane (CH_4_) through enteric fermentation. Recent advances in our understanding of methanogenesis have led to the development of animal feed additives (AFA) that can reduce enteric CH_4_ emissions. However, many interacting factors impact hydrogen (H_2_) and CH_4_ production and AFA efficacy, including animal factors, basal diet, particle and fluid outflow, microbial populations, rumen fluid pH, and fermentative cofactor dynamics. Characterizing the response of rumen fermentation to AFA is essential for optimizing AFA implementation. Mechanistic models of enteric fermentation are constructed to represent physiological and microbial processes in the rumen and can be updated to characterize the dependency of AFA efficacy on basal diet and the impacts of AFA on fermentation. The objective of this article is to review the current state of rumen mechanistic modeling, contrasting the representation of key pools in extant models with a particular emphasis on representation of CH_4_ production. Additionally, we discuss the first rumen mechanistic models to include AFA and emphasize future model needs for improved representation of rumen dynamics under CH_4_-inhibition due to AFA supplementation, including the representation of microbial populations, rumen pH, fractional outflow rates, and thermodynamic control of fermentative pathways.

## 1 Introduction

Due to the importance of ruminants in the global food supply, developing quantitative approaches to optimizing production has been a major focus of ruminant nutritionists. Recently, attention has shifted to using quantitative methods to minimize environmental impacts of ruminant production (Dijkstra et al., 2005). Livestock agriculture is responsible for the direct production of greenhouse gasses (GHG) such as methane (CH_4_) through enteric fermentation and nitrous oxide (N_2_O) and CH_4_ from manure management, as well as indirect GHG production associated with feed production and conversion of forest into pasture (Caro et al., 2016).

Enteric fermentation is the digestive process by which feed is broken down by microorganisms in the rumen. This process uniquely allows ruminants to utilize fibrous plants as energy sources (Krehbiel, 2014). While CH_4_ is considered a loss of 2–10% of ingested gross energy (GE) (Moe and Tyrrell, 1979), methanogenesis is a vital sink of reducing equivalents which, without disposal, could potentially inhibit the reoxidation of microbial cofactors and depress fermentation (Morgavi et al., 2010). Microbes in the rumen ferment carbohydrates to volatile fatty acids (VFA), the major energy source for ruminant hosts, as well as carbon dioxide (CO_2_) and hydrogen (H_2_). Archaea in the rumen then perform methanogenesis by utilizing several metabolic pathways to reduce substrates such as CO_2_ with H_2_ to form CH_4_ (Hook et al., 2010; Smith and Hungate, 1958).

Recently developed animal feed additives (AFA) can reduce enteric CH_4_ emissions by directly disrupting methanogenesis or modifying the rumen environment to promote alternative metabolic pathways (Honan et al., 2021). However, many interacting factors impact H_2_ and CH_4_ production, such as fractional outflow rates, microbial populations, and microbial cofactor dynamics. In addition, basal diet, animal factors such as cattle type, body weight, feed intake, and their interactions affect AFA efficacy (Dijkstra et al., 2018; Kebreab et al., 2023). To optimize AFA implementation, it is essential to characterize the response of rumen fermentation to these additives. However, this task is laborious when studied *in vivo* and challenging using empirical models, which do not account for complex interactions between variables. In contrast, mechanistic models are constructed to represent physiological processes. While the complexity of mechanistic models and their dependence on parameters that are difficult to obtain can make their use impractical in some settings (Ross et al., 2024), they can nonetheless be valuable research tools to understand the dependency of AFA efficacy on rumen parameters and optimize AFA implementation.

Several dynamic, mechanistic models of rumen fermentation have been developed, but few explicitly represent AFA. Bannink and De Visser (1997) offered a quantitative comparison of several of these models and Ellis et al. (2008) surveyed microbial factors salient to mechanistic rumen modeling. Kebreab et al. (2009) reviewed both mechanistic and empirical models of nutrient excretion by ruminants and Bannink et al. (2016) reviewed how mathematical modeling contributes to understanding rumen fermentation. However, none of these articles thoroughly review the mathematical representations of microbial elements in extant rumen models, nor do they discuss these elements under conditions of CH_4_-inhibition. The recent advent of molecular methods has allowed deeper characterization rumen microbial communities, including under CH_4_-inhibition (Indugu et al., 2024; Zhao et al., 2024), that was not previously available for incorporation into mechanistic models. In addition, data from *in vitro* studies characterizing the rumen microbiome, including its response to AFA, have been incorporated into and used to evaluate predictions and identify influential parameters in *in vitro* fermentation models (Blondiaux et al., 2024; Merk et al., 2023; Muñoz-Tamayo et al., 2021). Thus, *in vitro* studies and models are important steps toward incorporating AFA into full rumen models. Revisiting the current state of rumen mechanistic models considering these recent advances is necessary.

The objective of this article is to review current mechanistic models of rumen fermentation and their representations of rumen fermentation via state variables and control elements, with specific focus on those capable of predicting enteric CH_4_ emissions. We focus predominantly on comprehensive models of rumen fermentation, but also discuss specialized models focusing on particular aspects of rumen fermentation such as lipid biohydrogenation, starch degradation, and rumen outflow. We begin with an overview of the historical development of rumen mechanistic models and typical model structures and then review representations of rumen state variables in these models, emphasizing updated model needs specifically for modeling rumen fermentation and CH_4_ production under AFA supplementation.

## 2 Mechanistic model structure

Classifying a mathematical model as “mechanistic” denotes that it predicts the behavior of a system by simulating elements of the system at a lower level of aggregation than the system itself, such as simulating the behavior of rumen microbes to predict rumen function. The models of rumen fermentation discussed here are structured according to the rate: state formalism. In this formalism, a “state variable” is a biological entity that determines the state of the system and state variable quantities are called “pools.” The system to be modeled is defined in terms of state variable pools and the rates of exchange between these pools (Thornley and France, 2007). Transactions of state variables from one pool to another (“fluxes”) are catalyzed by enzymes and can be represented using enzyme kinetic equations including mass action, Michaelis–Menten, sigmoidal (allosteric), and inhibitory kinetics (Gill et al., 1989). General forms of these flux equations are given in Table 1, Panel B. Subtracting the sum of all outputs from a state variable pool from the sum of all its inputs gives a first-order ordinary differential equation (ODE) for each state variable (Panel C). Thus, dynamic mechanistic models consist of a system of ODE describing the rate at which state variable pools change over time (Panel A). For a simplified example, see Panel D. The mechanistic model is then typically run by numerically integrating each ODE to give state variable pool size at each timestep, given initial pool size conditions.

**Table 1:** Mechanistic model notation and general equation forms.

**A. Mechanistic model: a system of differential equations**A mechanistic model is a system of differential equations given as: $dQ_{1}/dt=f_{1}\left(Q_{1},\;Q_{2},\;Q_{3},\ldots ,Q_{n}\right)$ $dQ_{2}/dt=f_{2}\left(Q_{1},\;Q_{2},\;Q_{3},\ldots ,Q_{n}\right)$ *…* $dQ_{n}/dt=f_{n}\left(Q_{1},Q_{2},Q_{3},\ldots ,Q_{n}\right)$ where *dQ_n_/dt* represents the change in pool *Q_n_* with respect to time and *f_n_* represents some function of the pools of the state variables.
**B. General forms of kinetic flux equations** Mass action: $v=k\left[S_{1}\right]N1$ for systems with a single substrate $v=k{\left[S_{1}\right]}^{N1}{\left[S_{2}\right]}^{N2}\ldots {\left[S_{n}\right]}^{Nn}$ in a system with multiple substrates, where *v* is the reaction velocity, *k* is the mass action constant, *S* are substrates. Michaelis–Menten and allosteric/inhibitory relationships: $v=V_{\text{max}}/(1+{\left(K_{1}/\left[S_{1}\right]\right)}^{N1}+\ldots +{\left(K_{n}/\left[S_{n}\right]\right)}^{Nn}+{\left(\left[I_{1}\right]/J_{1}\right)}^{M1}+\ldots +[{\left(I_{n}]/J_{n}\right)}^{Mn})$ or $v=V_{\text{max}}/\left(1+{\left(K_{1}/\left[S_{1}\right]\right)}^{N1}\right)\times \ldots \times \;\left(1+{\left(K_{n}/\left[S_{n}\right]\right)}^{Nn}\right)\times$ $\left(1+{\left(\left[I_{1}\right]/J_{1}\right)}^{M1}\right)\times \ldots \times \;\left(1+{\left(\left[I_{n}\right]/J_{n}\right)}^{Mn}\right)$ where *V_max_* is the maximum reaction velocity, *S* are substrates with allosteric interactions, *K* are Michaelis–Menten constants corresponding to S, *I* are inhibitors, *J* are inhibition constants corresponding to *I,* and *N* and *M* are steepness parameters.
**C. Differential equation for the state variable pool QiKinetic flux equations can be summed to give a differential equation for each state variable pool as:** $dQ_{i}/dt=\Sigma P_{i,jm}-\Sigma U_{i,jm}$ where input fluxes to and output fluxes from pools are denoted *P_i,jm_* and *U_i,jm_*, respectively, where the subscript represents the uptake (U) or production (P) of pool i by j-to-m transaction.
**D. Simplified example** Influx of fiber (Fb) into the rumen can be modeled as: $P_{Fb,\mathrm{Intake}-Fb}\;\left(\mathrm{grams}\;Fb/h\right)=\mathrm{Feed}\;\mathrm{intake}\;\mathrm{rate}\;\left(g/h\right)\times \;\mathrm{feed}\;\mathrm{fiber}\;\mathrm{concentration}\;\left(g\;Fb/g\;\mathrm{feed}\right)$ Outflow of fiber from the rumen occurs due to hydrolysis into hexoses (He): $U_{Fb,Fb-\mathrm{He}}=Q_{Fb}\times \;\mathrm{fiber}\;\mathrm{hydrolysis}\;\mathrm{rate}$ Correspondingly, one of many inflows to the He pool is fiber hydrolysis: *P_He, Fb-He_ = U_Fb, Fb-He_*. The differential equation describing the change in degradable fiber pool size is thus defined as: *dQ_Fb_/dt = ΣP_Fb_ - ΣU_Fb_*.

Adapted from Gill et al. (1989), Thornley and France (2007), and van Lingen et al. (2019). Additive or multiplicative Michaelis–Menten equations in Panel (B) can be selected based on the nature of substrate–substrate or substrate-inhibitor interactions (e.g., competitive or non-competitive inhibition). See Thornley and France (2007) for more complete discussion of equation forms. Square brackets denote concentrations of substrates or inhibitors.

## 3 Overview of historical development of rumen nutritional models

Mechanistic models from five major “lineages” are discussed in this review. These “lineages” were selected because the original models and their successors encompass the most significant efforts in modeling whole-rumen function mechanistically. The earliest model discussed here is Baldwin et al. (1977) along with related models (Argyle and Baldwin, 1988; Baldwin, 1995; Baldwin et al., 1987; Reichl and Baldwin, 1975), collectively known as MOLLY. MOLLY has been updated in several models (e.g., Gregorini et al., 2015; Vetharaniam et al., 2015). A separate “lineage” is represented by France et al. (1982). A third “lineage” is represented by Dijkstra et al. (1992), which was later updated to improve representation of protozoa by Dijkstra (1994). These models are known as COWPOLL. Several subsequent models were based on COWPOLL such as Dijkstra et al. (1996b) and Mills et al. (2001, 2014). van Lingen et al. (2019, 2021) are closely related to COWPOLL but do not directly update it. Another “lineage” is the Karoline model (Danfær et al., 2006; Huhtanen et al., 2015; Ramin and Huhtanen, 2015). Some overlap of “lineages” exists as elements of models in one lineage were incorporated into others. More recently, Muñoz-Tamayo et al. (2016) developed a model of *in vitro* fermentation which utilizes a novel representation of microbial functional groups; this model was later updated in Muñoz-Tamayo et al. (2021), representing a fifth “lineage.” While Muñoz-Tamayo et al. (2016, 2021) are *in vitro* models, they introduce elements relevant to the objectives of this review and are discussed here. Other models that are more specialized to represent aspects of rumen fermentation in more detail are also discussed. Table 2 summarizes salient control features, with a particular emphasis on modeling CH_4_ production, of the comprehensive rumen models included in this review.

**Table 2:** Overview of representations (rep.) of key microbial and nutritional elements included (incl.) in main mechanistic models of rumen fermentation discussed in this review.

Model	“Predecessor” or related model(s)	CH_4_ rep.	AFA incl.	VFA incl.	Microbial groups incl.	Fractional rumen outflow rep.	Thermodynamic control	Other controls, e.g., pH, intake pattern, etc.
France et al. (1982)	No direct predecessor	None	None	None	General microbes	Mechanistically modeled based on rumen fluid pool size	None	Pulsed feed inputs to simulate non-continuous feed intake
Baldwin (1995)	Argyle and Baldwin (1988), Baldwin et al. (1977, 1987)	H_2_ balance	None	Acetate (Ac), propionate (Pr), butyrate (Bu), lactate (La)	General microbes subdivided by association with particles of different sizes	Particle-size based	None	pH estimated empirically based on relative proportions of VFA and lactate; whole animal model with digestion and metabolism sub-models
Dijkstra (1994)	Dijkstra et al. (1992)	None	None	Ac, Pr, Bu, valerate (Vl)	Amylolytic (Ba) and fibrolytic bacteria (Bf), protozoa (Po)	Constant solid and liquid outflow rates	None	pH, time below critical pH, and minimum pH specified by user
Mills et al. (2001)	Dijkstra et al. (1992)	H_2_ balance	None	Ac, Pr, Bu, Vl	Amylolytic and fibrolytic microbes	Constant solid and liquid outflow rates	None	pH estimated empirically based on VFA concentrations
Danfær et al. (2006)	Huhtanen et al. (2015), Ramin and Huhtanen (2015)	Stoichiometric fermentation coefficients	None	Ac, Pr, Bu, branched chain VFA	General microbes disaggregated by microbial composition	General passage rate modified to be more specific for many components	None	Whole animal model with digestion and metabolism sub-models
Mills et al. (2014)	Dijkstra (1994)	None	None	Ac, Pr, Bu, La	Ba with sub-groups of lactate-utilizers and lactate-producers, Bf, Po	Constant solid and liquid outflow rates	None	pH estimated empirically based on lactate and VFA concentrations; pulsed dietary inputs
Van Lingen et al. (2019)	No direct predecessor but related to COWPOLL	Based on kinetic H_2_ uptake for methanogen growth	None	Ac, Pr, Bu	General microbes, methanogens (Me)	Constant solid and liquid outflow rates	Thermodynamic control of fermentation pathways via NAD+/NADH ratio, controlled by pH_2_	Pulsed feed inputs to simulate non-continuous feed intake
van Lingen et al. (2021)	van Lingen et al. (2019)	Based on kinetic H_2_ uptake for methanogen growth	3NOP, nitrate, 3NOP- and nitrate-metabolite nitrite	Ac, Pr, Bu	General microbes, Me	Constant solid and liquid outflow rates	Thermodynamic control of fermentation pathways via NAD+/NADH ratio, controlled by pH_2_	Pulsed feed inputs to simulate non-continuous feed intake
Muñoz-Tamayo et al. (2016)	No direct predecessor	Based on kinetic H_2_ uptake for H_2_-utilizer growth	None	Ac, Pr, Bu	Sugars-, amino acids-, and hydrogen-utilizing microbes	No outflow (*in vitro* model)	None	Mechanistic pH based on charge balance
Muñoz-Tamayo et al. (2021)	Muñoz-Tamayo et al. (2016)	Based on kinetic H_2_ uptake for H_2_-utilizer growth	Bromoform from *A. taxiformis*	Ac, Pr, Bu	Sugars-, amino acids-, and hydrogen-utilizing microbes	No outflow (*in vitro* model)	pH_2_ controls VFA flux allocation; implicitly assumes linearity between pH_2_ and NADH/NAD+	Mechanistic pH based on charge balance

In the case of several models developed in the same “lineage,” the later model is presented, but subsequent models that introduce wholly new aspects are presented separately. See text for more detailed discussion of each aspect.

## 4 Review of state variable pools in current rumen mechanistic models

### 4.1 Feed fractions

The primary goal of mechanistic models of rumen fermentation is to mathematically describe the microbial transactions that transform feed into metabolites, including CH_4_. Consequently, most models incorporate detailed representation of feed fractions, especially carbohydrates and nitrogen (N) sources. Below, we review the representation of feed fraction categories in extant rumen models. Most models include dietary inputs into each feed fraction as a continuous intake rate (Baldwin, 1995; Dijkstra, 1994; Dijkstra et al., 1992). However, some models can represent pulsed dietary inputs to simulate non-continuous feeding patterns (France et al., 1982; van Lingen et al., 2019).

#### 4.1.1 Carbohydrates

Carbohydrate feed fractions are generally divided into at least four fractions: degradable starch, degradable fiber, undegradable fiber, and soluble carbohydrates (Nozière et al., 2010). Below is a detailed review of how carbohydrate fractions are represented in various models.

##### 4.1.1.1 Fiber

**Figure 1:**
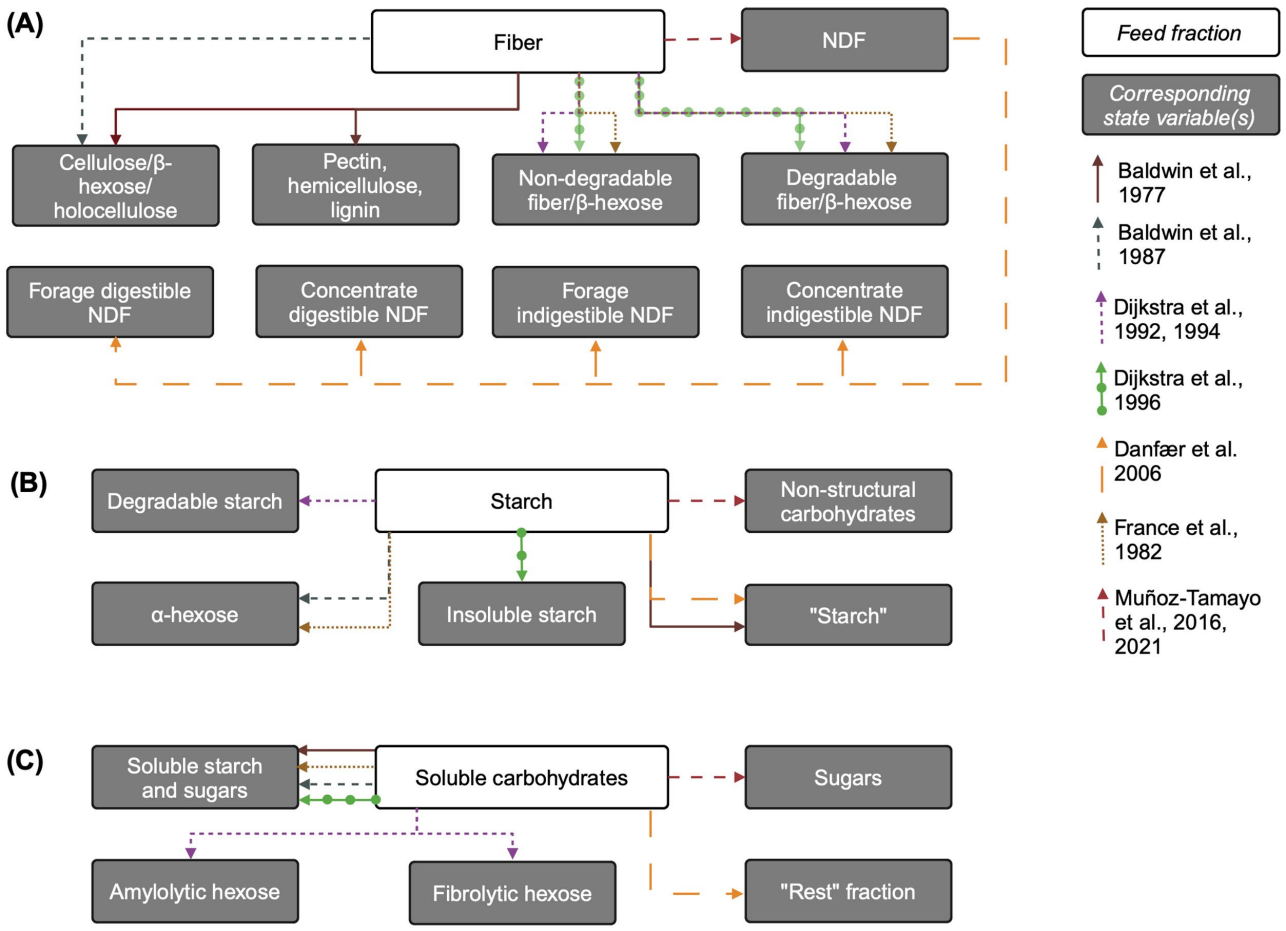
Overview of carbohydrate feed fractions and their corresponding state variables in mechanistic rumen fermentation models. (A) Fiber. (B) Starch. (C) Soluble carbohydrates. The biological feed fraction entity is linked to its corresponding state variable via an arrow corresponding to each model according to the key on the right of the figure. Some state variable pools are aggregated for simplicity. When the state variable name is the same as the biological entity, the state variable name is written in quotation marks. Associations of insoluble state variable pools with particles is not shown. The “Rest” fraction in Danfær et al. (2006) refers to sugars and other organic matter not accounted for. Created in BioRender. Pressman, E. (2024a) https://BioRender.com/z67x384.

See Figure 1A for an overview of state variables corresponding to fiber included in models reviewed here. Baldwin et al. (1977) explicitly represents plant insoluble carbohydrates such as pectin, hemicellulose, cellulose, and lignin. These fractions are further disaggregated into available (non-solubilized), fluid-associated, and fluid-and particle-associated-microbe-associated pools. This complex representation is simplified by Baldwin et al. (1987), which combines hemicellulose and cellulose into holocellulose (also referred to as *β*-hexose), and includes lignin and insoluble ash. Cell wall content (hemicellulose, cellulose, and lignin) is used to scale rumination rate in Baldwin et al. (1987).

Subsequent rumen mechanistic models often use a more aggregated and functional approach, assuming that non-structural plant carbohydrates (e.g., starch) are fully degradable while structural carbohydrates contain both degradable and non-degradable fractions. The two latter fractions are found in most models. Structural carbohydrates are represented by “non-rumen-degradable β-hexose” and “rumen-degradable β-hexose” in France et al. (1982) and “degradable” and “undegradable fiber” pools in Dijkstra (1994) and Dijkstra et al. (1992, 1996b). A more recent model uses neutral detergent fiber (NDF) as the state variable pool representing structural carbohydrates (Muñoz-Tamayo et al., 2016). Danfær et al. (2006) uses a hybrid approach between functional and chemical classification, whereby the only fiber input is NDF, which is subdivided into forage and concentrate NDF and further divided into digestible and indigestible forage and concentrate NDF, respectively.

Inputs to the “fiber” pool come only from the diet. Some models associate fiber with a large particle pool (Baldwin et al., 1977, 1987) and Danfær et al. (2006) aggregates NDF, starch, and sugars pools into escapable and non-escapable carbohydrate pools, where the non-escapable pool corresponds to large particles. The allocation of structural carbohydrate chemical entities into these pools links fiber chemical structure with model representation. For example, in Baldwin et al. (1987), hemicellulose and cellulose (not themselves state variables) comprise the dietary input to the holocellulose pool. In Dijkstra et al. (1996a), undegradable fiber is the percent of NDF in the diet undigested after prolonged rumen incubation, multiplied by NDF, while undegradable fiber is NDF minus degradable fiber (Tamminga et al., 1990). Baldwin et al. (1987) includes degradable fiber in three separate pools of differing particle sizes.

Outputs from the degradable fiber pool in Dijkstra (1994) and Dijkstra et al. (1992) include only hydrolysis by fibrolytic microbes to “fibrolytic hexose” (a pool of hexose only accessible to fibrolytic bacteria) and outflow from the rumen with the solid fraction. Typically, fiber hydrolysis is represented using mass action kinetics with a static fiber hydrolysis rate (Muñoz-Tamayo et al., 2016); mass-action with dependence on cellulolytic microbial mass is also common (e.g., Baldwin et al., 1987; France et al., 1982; van Lingen et al., 2019). In Dijkstra (1994) and Dijkstra et al. (1992), fiber hydrolysis is represented using Michaelis–Menten kinetics, dependent on cellulolytic microbial mass, and is sigmoidally regulated by rumen pH, with the hydrolysis rate declining at lower pH levels. In Baldwin et al. (1987), the holocellulose hydrolysis rate is dependent on the mass of microbes associated with holocellulose, and Argyle and Baldwin (1988) updated this representation to include pH effects on fiber hydrolysis. Non-escapable carbohydrates in Danfær et al. (2006) are either fermented or released to the escapable pool, whereby the fermentation rate decreases with increasing diet starch and sugars content, implicitly representing dependence on the fibrolytic bacterial population in a less mechanistic manner, as liquid-associated microbe population depends on the ratio of soluble carbohydrates to total NDF in the diet. Escapable carbohydrates are either fermented or flow out to the small intestine.

The fiber hydrolysis rate is influential on CH_4_ predictions. An error of 1%/hour in the NDF degradation rate in Huhtanen et al. (2015) would cause a 2.4% error in CH_4_ predictions, and the fiber hydrolysis rate in van Lingen et al. (2019) accounted for 6.2% of variation in predicted CH_4_ output, making it the fourth largest contributor to CH_4_ uncertainty among model parameters. The authors noted that this result agreed with empirical CH_4_ prediction equations, where fibrous fractions, unlike starch and sugars, typically appear (Appuhamy et al., 2016). They hypothesized that a more mechanistic representation of the fiber hydrolysis rate (e.g., by representing its dependence on pH or on feed particle size) might improve CH_4_ predictions. However, Muñoz-Tamayo et al. (2016) found that in their model of *in vitro* batch fermentation, the fiber hydrolysis rate constant was similar across all four experimental diet scenarios simulated. This suggests that more mechanistic representation of the fiber hydrolysis rate would not explain differences in CH_4_ production across experiments. This may reflect the particularly influential role of fiber hydrolysis rate in terms of its interplay with particulate outflow from the rumen in a model of *in vivo* fermentation. These results suggest that, at least in the *in vivo* modeling scenario, more mechanistic control of fiber hydrolysis rate and solid outflow rate may improve CH_4_ predictions.

##### 4.1.1.2 Starch

Mechanistic models generally assume that all insoluble starch is degradable and represent it using a “degradable starch” state variable (Dijkstra, 1994; Dijkstra et al., 1992), “insoluble starch” (Dijkstra et al., 1996b), “*α*-hexose” (Baldwin et al., 1987; France et al., 1982), simply “starch” (Baldwin et al., 1977; Danfær et al., 2006), or the more general “non-structural carbohydrates” (Muñoz-Tamayo et al., 2016) (Figure 1B). In conceptual models of starch degradation, Nocek and Tamminga (1991) represent directly soluble starch, and Beever et al. (1993) conceptualize starch fractions as potentially degradable or undegradable, with potentially degradable split into that actually degraded to hexose and that passing out of rumen (Mills et al., 1999). Additional complexity is added to starch representation by models which describe digested nutrients in terms of particle size distribution (Baldwin et al., 1987; Gregorini et al., 2015).

Inputs to the insoluble starch pool include only dietary inputs. In models that represent microbial storage polysaccharides (e.g., glycogen) (Dijkstra, 1994; Dijkstra et al., 1992), these are represented as separate state variables that do not enter the starch pool, but instead the soluble carbohydrates pool upon microbial lysis. However, in Muñoz-Tamayo et al. (2016), the recycling of microbial cells is included as an input to the starch pool.

Outputs from the starch state variable pool generally include hydrolysis either to a single pool containing soluble starch and sugars (Dijkstra et al., 1996b; France et al., 1982; Muñoz-Tamayo et al., 2016) or hydrolysis by amylolytic microbes to amylolytic hexose (Dijkstra, 1994; Dijkstra et al., 1992). In the model with the most complex representation of microbial metabolism and explicit representation of protozoa, insoluble starch is directly taken up by protozoa for both growth and storage polysaccharide formation (Dijkstra, 1994). In Dijkstra (1994), protozoal uptake of starch is represented using saturation kinetics, with allosteric inhibition by the intracellular storage polysaccharide content of protozoa. Most models represent insoluble starch outflow from the rumen with the solid fraction (Baldwin et al., 1987; Dijkstra, 1994; Dijkstra et al., 1992, 1996b). Baldwin et al. (1987) and France et al. (1982) use α-and *β*-hexose-specific outflow rates. Like fiber, starch hydrolysis is generally represented with first-order or Michaelis–Menten kinetics.

Starch-related state variables are generally represented with similar or less complexity and fewer state variables than fiber. This may reflect that starch is a relatively more homogenous chemical entity than fiber, although few mechanistic models attempt to represent starch in terms of starch granule structure or the degree and type of starch processing. Mills et al. (1999) reviewed representations of starch degradation in rumen mechanistic models and recommend that starch model inputs be characterized in terms of degradability, as well as physical form of starch and proportions of large, small and soluble fractions. However, such characterizations may be difficult to define in terms of proximate analysis fractions as with fiber. Mechanistic models developed since then have not introduced major changes in starch representations. Improved starch description may facilitate improved representation of carbon and redox balances (Baldwin, 1995; Mills et al., 1999), which may gain more importance as thermodynamic control of CH_4_ production is increasingly considered.

##### 4.1.1.3 Soluble carbohydrates and hexoses

The products of complex carbohydrate hydrolysis are often represented as a single pool containing various soluble sugars, or as multiple pools disaggregated by the microbial groups they are accessible to (Figure 1C). Sugars are included in a generalized “soluble starch and sugars” pool in Baldwin et al. (1987) and Dijkstra et al. (1996b) and in the “Rest” fraction in Danfær et al. (2006), which represents sugars and all other organic matter unaccounted for. In Dijkstra et al. (1996b), the sugars pool includes dietary water-soluble carbohydrates, glycerol from the hydrolysis of long chain fatty acids, and the hydrolysis products of degradable fiber or insoluble starch, whereas in Danfær et al. (2006), glycerol from fatty acids have its own pool. Similarly, Baldwin et al. (1987) and France et al. (1982) include hydrolysis of the structural carbohydrates as inputs to the soluble sugars pool. France et al. (1982) also accounts for the release of water-soluble carbohydrates via microbial catabolism.

Dijkstra (1994) and Dijkstra et al. (1992) offer a more complex representation of microbial metabolism, disaggregating water-soluble carbohydrates into amylolytic and fibrolytic hexoses. These hexoses, produced through the hydrolysis of insoluble starch and fiber, respectively, are available only to the corresponding microbial group. Microbial catabolism serves as a source of amylolytic hexose and in Dijkstra et al. (1992) the death and lysis of amylolytic microbes is a source of amylolytic hexose. The Dijkstra (1994) model further distinguishes between amylolytic bacteria and protozoa, with additional sources of amylolytic hexose arising from glycerol (from dietary and protozoal lipid) and hydrolyzed protozoal storage polysaccharides released through protozoal lysis. Finally, lactate is included in the amylolytic hexose pool in both Dijkstra models and as a soluble carbohydrate in Danfær et al. (2006). In contrast, Muñoz-Tamayo et al. (2016) uses a simpler representation, where a “sugars” pool is produced solely from the hydrolysis of structural and non-structural carbohydrates.

Several models (Baldwin et al., 1987; Dijkstra, 1994; Dijkstra et al., 1992, 1996b; France et al., 1982) include the uptake of water soluble carbohydrates for microbial growth on ammonia and soluble protein and for non-growth purposes. These uptakes are disaggregated by microbe type in the Dijkstra models. Outputs from each hexose pool in Dijkstra et al. (1992) include microbial growth with ammonia, growth with soluble protein, utilization for non-growth, and outflow. Dijkstra (1994) updated this model to include engulfment by protozoa as an output of fibrolytic hexose, and in this model, amylolytic hexose is also taken up for protozoal growth and storage polysaccharide formation by amylolytic bacteria and protozoa. Muñoz-Tamayo et al. (2016) includes uptake for fermentation as the sole sugar utilization pathway.

As hexose is a key substrate for microbial growth and VFA production, the representation of water-soluble carbohydrates can considerably influence the dynamics of microbial growth and substrate utilization. Baldwin et al. (1987) was the first model to introduce microbial substrate with distinct amylolytic and fibrolytic hexose groups, a strategy adopted by subsequent models to prevent biologically inappropriate interactions. However, in mixed cultures, interspecies cross feeding occurs, where microbial species utilize products of other species’ digestive enzymes (Krause et al., 2013). Baldwin (1995) argued that data were insufficient to represent and parameterize these complex interactions at that time. While cross-feeding of hexoses remains underrepresented in more recent rumen fermentation models, Mills et al. (2014) includes uptakes by one microbial group of lactate produced by another group. Given the importance of cross-feeding in the digestion of plant carbohydrates, incorporating representations of microbial cross-feeding may improve predictions of carbohydrate degradation and potentially CH_4_ formation.

#### 4.1.2 Protein/growth substrates

**Figure 2:**
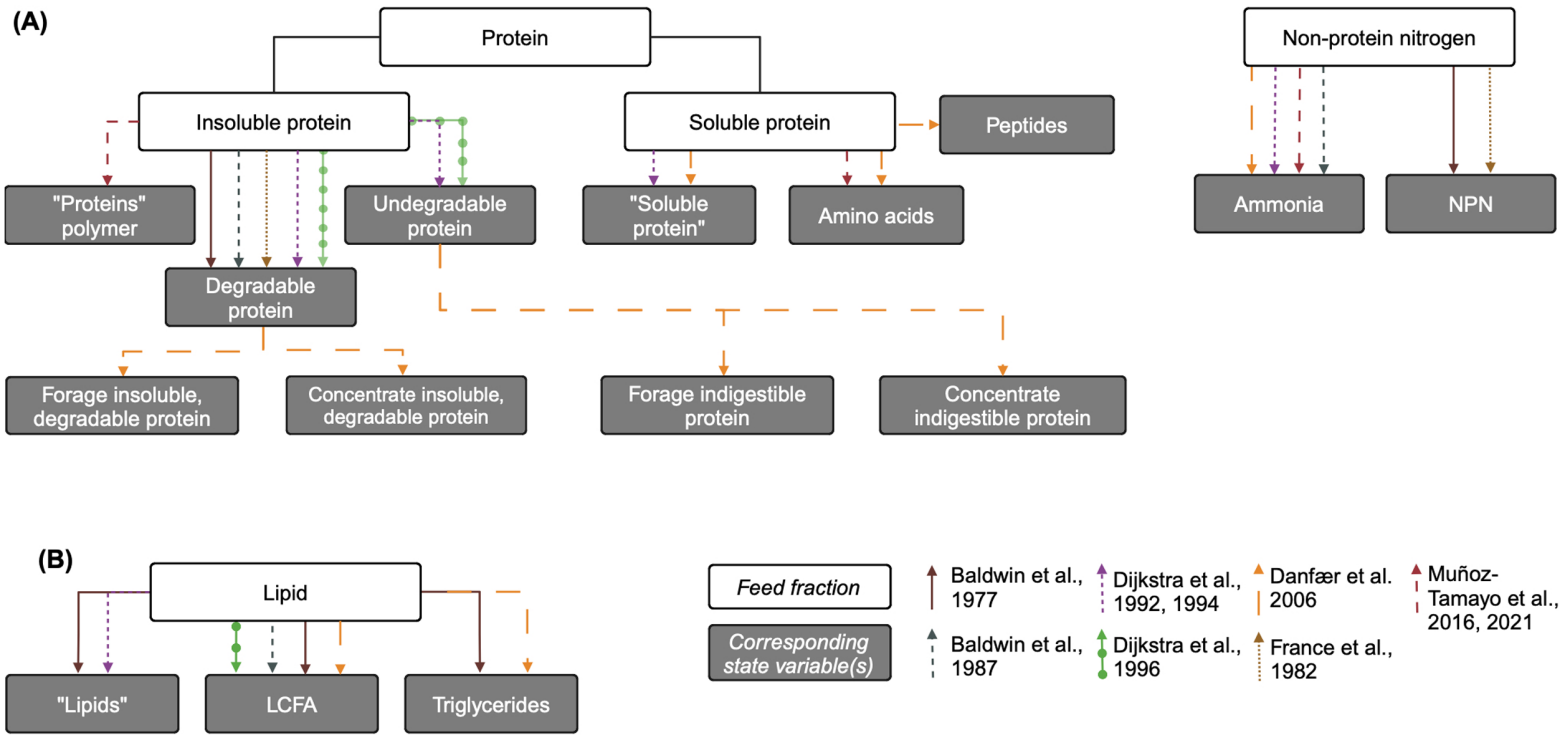
Overview of protein and lipid feed fractions and their corresponding state variables in mechanistic rumen fermentation models. (A) Protein and non-protein nitrogen. (B) Lipids. The biological feed fraction entity is linked to its corresponding state variable via an arrow corresponding to each model according to the key on the bottom right of the figure. Some state variable pools are aggregated for simplicity. When the state variable name is the same as the biological entity, the state variable name is written in quotation marks. LCFA: long chain fatty acid. Created in BioRender. Pressman, E. (2024b) https://BioRender.com/e11l750.

Nitrogenous feed fractions are generally disaggregated into at least two fractions in complex rumen models: soluble protein (amino acids) and non-protein nitrogen (NPN; ammonia and/or urea). Most models also include insoluble protein that is hydrolyzed to soluble protein, and fewer models include an undegradable protein fraction. The representations of growth substrates in each model are reviewed below and an overview is presented in Figure 2A.

##### 4.1.2.1 Undegradable protein

Some models represent the variable digestibility of insoluble protein by including an undegradable protein pool, while others assume that all protein is eventually degraded in the rumen. Dijkstra (1994) and Dijkstra et al. (1992, 1996b) include an undegradable protein pool whose only source is the feed, with outflow as its only uptake. In Dijkstra et al. (1996a), undegradable protein is defined as the percent of crude protein undigested after prolonged rumen incubation, multiplied by crude protein content (Tamminga et al., 1990). Similarly, “totally indigestible” forage and concentrate protein and “non-rumen-degradable protein” pools are included in Danfær et al. (2006) and France et al. (1982), respectively, with dietary input and outflow as their only fluxes.

While these undegradable protein pools do not interact with other pools, they may impact nutrient availability. Modifying Baldwin et al. (1987) to include undegradable protein and NDF pools led to reduced rumen availability of NDF and protein, which in turn decreased digestion and microbial growth (Bannink and De Visser, 1997). Increasing the insoluble protein degradation rate in Danfær et al. (2006) reduced predicted CH_4_, but this effect was small. Subdividing protein pools based on digestibility, as is more common in fiber pools, may improve prediction of nutrient utilization.

##### 4.1.2.2 Degradable protein

In most models, feed protein enters as either insoluble protein or soluble protein (free amino acids), or into the undegradable protein pool if included. Without an undegradable protein pool, insoluble protein is considered completely degradable to free amino acids via microbial hydrolysis. A degradable protein pool is included in Baldwin et al. (1977), France et al. (1982), Baldwin et al. (1987), Dijkstra (1994) and Dijkstra et al. (1992, 1996b). Danfær et al. (2006) contains both forage and concentrate insoluble, degradable protein pools. Muñoz-Tamayo et al. (2016) includes a single protein polymer pool assumed to be degradable, functionally equivalent to a degradable insoluble protein pool. In Dijkstra et al. (1996a), the soluble protein fraction is defined as the protein fraction that washes out of nylon bags without rumen incubation, and the degradable protein pool is the dietary crude protein content, minus the soluble and undegradable protein fractions (Tamminga et al., 1990). However, in the aggregated protein polymer pool of Muñoz-Tamayo et al. (2016), this pool also includes recycled microbial cell protein, which other models include in the soluble protein pool.

Uptakes for insoluble protein include outflow with the solid fraction and hydrolysis to soluble protein in most models. In Dijkstra (1994), insoluble protein can be taken up directly by protozoal engulfment. In Baldwin et al. (1977, 1987), where insoluble feed particle size distributions are considered, the insoluble protein pool contributes to the large particle pool size, and only the portion of insoluble protein not associated with large particles is available for passage. Similarly to carbohydrates, insoluble protein pools (degradable and undegradable protein from forage and concentrate) are aggregated into inescapable nitrogen pools and are either fermented or released to the escapable nitrogen pool in Danfær et al. (2006).

Degradable (insoluble) protein hydrolysis is typically represented using modified mass action kinetics dependent on the relevant microbial pool size (Baldwin et al., 1977, 1987; Dijkstra et al., 1996b; France et al., 1982) or Michaelis–Menten kinetics (Dijkstra, 1994; Dijkstra et al., 1992). Most models do not explicitly include a proteolytic microbial group, so the microbial group relevant for protein hydrolysis is represented as the total microbial pool or, in Baldwin et al. (1987), the small-particle associated microbial pool. The models of Muñoz-Tamayo et al. (2016, 2021), however, explicitly represent an amino-acid utilizing bacterial pool.

##### 4.1.2.3 Soluble protein (amino acid protein)

Inputs to the soluble protein pool include the immediately soluble protein content of the feed, hydrolysis of insoluble protein, and salivary proteins. Models vary in the complexity of their representation of microbial protein recycling. The most complex representation is found in Dijkstra (1994), where microbial soluble protein inputs are disaggregated into those from the lysis of protozoa and the release of unutilized insoluble and bacterial protein engulfed by protozoa. Danfær et al. (2006) also utilizes a relatively complex representation of soluble protein, including separate pools for amino acids, soluble protein, and peptides. Inflow to the peptides pool is degradation of soluble protein, and inflows to amino acids are degraded peptides and recycled microbial protein. A more simple representation by Muñoz-Tamayo et al. (2016) only includes an aggregated input of “recycled microbial cell protein” into the protein polymer (not amino acid) pool.

Outputs from the soluble protein pool include outflow with the liquid fraction from the rumen and utilization by rumen microbes, the latter represented with varying complexity across models. Microbial amino acid uptakes for growth is typically modeled using either kinetic rates of substrate utilization or microbial growth rates, which are linearly related if maintenance requirements are negligible (Muñoz-Tamayo et al., 2016). Dijkstra (1994) disaggregates microbial soluble protein uptake into incorporation into amylolytic and fibrolytic microbial mass, fermentation to ammonia by these microbes, and uptake by protozoa. Dijkstra et al. (1996b) models soluble protein uptake for microbial growth using saturation kinetics dependent on soluble protein, energy (soluble carbohydrates), and microbial pool size, with soluble protein uptake for fermentation inhibited by soluble carbohydrates. Degraded amino acids are also inputs to the ammonia pool in Danfær et al. (2006). Baldwin et al. (1977) uses a more simplified representation of microbial amino acid uptake, where amino acids are used for microbial protein synthesis or fermentation disaggregated by free and attached microbes, with uptakes represented using mass-action kinetics. Baldwin et al. (1987) is similar, except that uptake for fermentation is represented using Michaelis–Menten kinetics. France et al. (1982) assumes that all rumen degradable protein is hydrolyzed to NPN, which is utilized by microbes for growth, with no direct uptake of degradable protein for growth. Muñoz-Tamayo et al. (2016) uses modified saturation kinetics for amino acid uptake, with dependence on the concentration of amino acids-utilizing microbes. The model of van Lingen et al. (2019) centers on carbohydrate and fermentative metabolism and does not include any N compounds.

##### 4.1.2.4 Ammonia

Many models include pools of NPN compounds (encompassing ammonia and less commonly urea and nucleic acids). France et al. (1982) includes an NPN pool from where all N utilized by microbes is taken up; inputs to this pool are salivary intake, degradation of rumen-degradable protein by proteolytic enzymes, and NPN released by microbial catabolism. Baldwin et al. (1977) includes nucleic acids and urea in an NPN pool, which solubilizes into an ammonia pool. Similarly, Baldwin et al. (1987) models NPN flow into the ammonia pool via the feed, amino acid fermentation, and saliva. Salivation rate is empirically modeled based on diet composition, and saliva urea concentration is assumed to be constant. Dijkstra (1994) and Dijkstra et al. (1992) include ammonia as a state variable. In Dijkstra (1994), inputs to the ammonia pool include feed ammonia content, urea transfer to rumen (modeled by Michaelis–Menten kinetics) and fermentation of soluble protein by amylolytic and fibrolytic microbes. Fermentation of protein engulfed by protozoa, including insoluble and bacterial and protozoal protein, also contributes to ammonia production. Danfær et al. (2006) also includes urea recycling, as well as amino acid degradation, as inputs to the ammonia pool.

Outflows from the ammonia pool include outflow with the liquid and utilization by microbes for growth on NPN, usually like equations for growth on soluble protein. Uptake of ammonia for growth by bacteria uses the same equation forms as those for soluble protein uptake for growth in Dijkstra (1994), but it is assumed that protozoa do not utilize ammonia. Unlike amino acids, most models include absorption of ammonia through the rumen wall. Dijkstra (1994) represents ammonia absorption using saturation kinetics dependent on the rumen surface area and pH. Baldwin et al. (1977) models ammonia utilization for microbial growth using mass-action process with stoichiometric requirements for growth on NPN. Baldwin et al. (1987) combines ammonia outflow and absorption into a single mass-action equation, representing ammonia uptake for microbial growth similarly to Baldwin et al. (1977). Dijkstra et al. (1996b) models ammonia uptake for microbial growth using saturation kinetics dependent on soluble protein, energy, and microbial pool size. Muñoz-Tamayo et al. (2016) includes ammonium ion as a state variable for charge balancing and dynamic pH prediction, and ammonia is a nitrogen source for sugar-and H_2_-utilizing microbial groups.

Although physiological soluble substrate concentrations are generally far below microbial affinities, making mass-action forms equally appropriate, Michaelis–Menten kinetics can represent saturating concentrations of soluble nutrients immediately after feeding (Argyle and Baldwin, 1989; Baldwin, 1995). Baldwin et al. (1987) and the Dijkstra models use a Michaelis–Menten form for all equations describing utilization of soluble nutrients. The Monod equation is a mathematical model for microorganism growth with the same form as the Michaelis–Menten equation. While the theoretical interpretation of the Michaelis–Menten equation applied to microbial growth has been questioned (Liu, 2007), “Monod growth” remains a flexible and easily parameterized model for microbial growth in the rumen environment (Dijkstra et al., 2002), generally agreeing with empirical gas production profiles better than other growth models (Dhanoa et al., 2000). Michaelis–Menten/Monod and Hill-type equations also have biologically interpretable parameters (Gill et al., 1989), and the kinetic approach to modeling microbial growth avoids the need for explicit maintenance requirements unlike the Pirt equation. The validity of Pirt “constants” for microbial species has also been questioned (Van Bodegom, 2007), especially given the aggregation typical of microbial pools in rumen models. The variable maintenance energy requirements of individual bacterial species coupled with variability in growth rates due to energy spilling led Dijkstra (1994) and Dijkstra et al. (1992) to avoid explicit maintenance energy requirements, instead calculating total energy required for non-growth functions based on energy and N availability. Similarly, Muñoz-Tamayo et al. (2016) does not explicitly represent microbial maintenance, assuming the cell death rate encompasses maintenance.

#### 4.1.3 Lipids

Despite the impact of long chain fatty acids (LCFA) on fiber degradation and CH_4_ production via the biohydrogenation H_2_ sink, representations of lipid metabolism in mechanistic rumen are generally less complex than those of carbohydrates and N compounds (Figure 2B). In fact, they are not present at all in France et al. (1982). Dijkstra et al. (1992) includes a “lipids” pool, while Baldwin et al. (1987) and Dijkstra et al. (1996b) include a “LCFA” pool. In these models, fluxes to both pools include input from the diet and outflow from the rumen. Additionally, Baldwin et al. (1987) and Dijkstra et al. (1996b) account for the incorporation of LCFA into microbial lipid. Lipids are hydrolyzed to LCFA, which can then undergo biohydrogenation as in Baldwin et al. (1977), which has a relatively complex representation of lipids with “lipid,” triglycerides, and LCFA pools. Similarly, Danfær et al. (2006) includes a rumen fat pool which corresponds to dietary crude fat, assumed to be triglycerides, as well as a free fatty acids pool. Triglycerides pass to free fatty acids by lipolysis, are taken up by rumen microbes, and flow out of the rumen. Baldwin (1995) empirically represents inhibition of fiber hydrolysis by fat but this effect is not related to the degree of LCFA saturation (Dijkstra et al., 2000).

Dijkstra (1994) extends the limited representation of lipid metabolism in Dijkstra et al. (1992) by including an input to the lipid pool from the lysis of protozoa and the release of engulfed lipid not utilized for protozoal growth. It also includes the uptake of lipid by protozoa for growth. Dijkstra et al. (2000) further extends Dijkstra et al. (1992) to more comprehensively represent rumen lipid metabolism, including dietary lipids and both saturated and unsaturated free LCFA. An updated version of Baldwin et al. (1987) described in Benchaar et al. (1998) also includes the biohydrogenation of unsaturated fatty acids to calculate hydrogen balance in the rumen. Similarly, Mills et al. (2001) updated Dijkstra et al. (1992) to predict CH_4_ production via H_2_ balance, including biohydrogenation. However, neither of these updated models includes a LCFA pool, so biohydrogenation is represented as a lipid uptake with an empirical constant accounting for proportion of saturated fat in dietary lipid.

Some specialized models of rumen lipid metabolism focus on kinetically representing the processes of unsaturated fatty acid biohydrogenation and the production of specific fatty acids (Moate et al., 2008). However, extant rumen models incorporating CH_4_-inhibiting additives (Muñoz-Tamayo et al., 2021; van Lingen et al., 2021) do not include biohydrogenation. As it is recommended that models incorporate alternative H_2_ sinks in light of H_2_ redirection with inhibited methanogenesis (Morgavi et al., 2023), increasingly complex representation of rumen lipid metabolism should become more common in rumen mechanistic models.

### 4.2 Microbe and microbial storage polysaccharide pools

**Figure 3:**
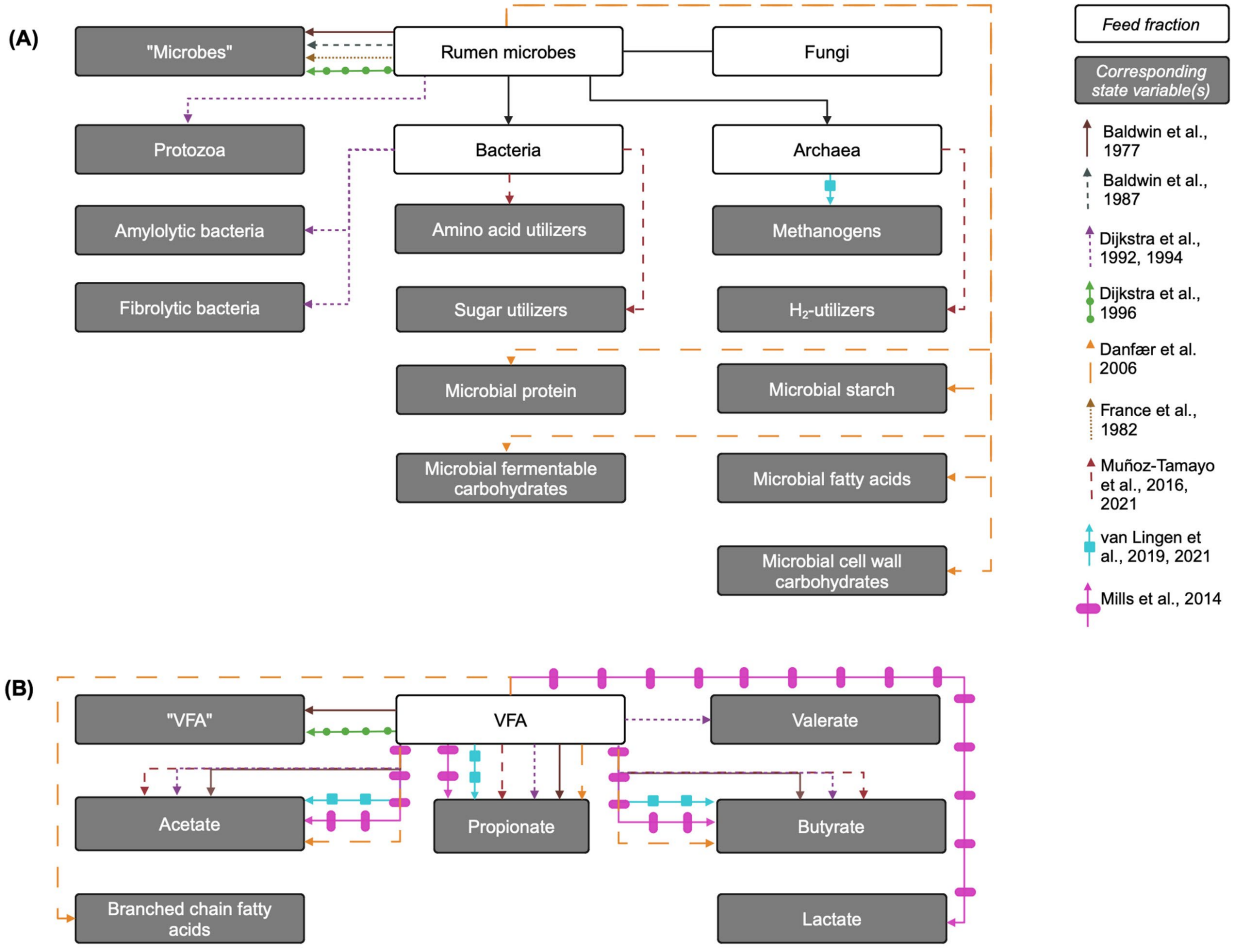
Overview of rumen microbe and fermentation products and their corresponding state variables in mechanistic rumen fermentation models. (A) Rumen microbial populations or components. (B) Fermentation products. The biological entity is linked to its corresponding state variable via an arrow corresponding to each model according to the key on the right of the figure. When the state variable name is the same as the biological entity, the state variable name is written in quotation marks. VFA: volatile fatty acids. Created in BioRender. Pressman, E. (2024c) https://BioRender.com/f30w782.

Representations of microbial groups vary in complexity across models, from single, aggregated pools of “microbes” to multiple bacterial sub-groups, protozoa, and methanogens. Distinctions within populations are generally made for bacteria according to carbohydrate utilization, although some models use alternative groupings (Muñoz-Tamayo et al., 2016). Many models assume a uniform and constant microbial dry matter composition (France et al., 1982), while others represent microbial storage polysaccharides or other microbial components as separate pools to account for their variable contributions (Danfær et al., 2006; Dijkstra et al., 1992). Below, representations of fermentative microbial subgroup in models are reviewed. The representations of methanogens are discussed in Section 4.5.3. See Figure 3A for an overview of state variables corresponding to microbial groups in each model reviewed here.

#### 4.2.1 Bacteria or aggregated fermentative microbes and bacterial storage polysaccharides

The simplest representation of fermentative microbes included in mechanistic rumen models is a single aggregated pool of microbes. This approach is utilized in Baldwin et al. (1977, 1987), Dijkstra et al. (1996b), and France et al. (1982). In the Baldwin models, the aggregated microbe pool is distributed across particle pools of different sizes, where microbes associated with different particle sizes access different substrates. This distribution implicitly distinguishes microbes based on substrate utilization. In a different approach, Danfær et al. (2006) models one microbial population that is disaggregated into pools of microbial components (microbial protein, starch, etc.), but uses one maintenance requirement that encompasses both amylolytic and fibrolytic bacteria. In contrast, Dijkstra et al. (1992) disaggregates microbes into amylolytic and fibrolytic microbial pools, where the amylolytic pool encompasses both bacteria and protozoa. Dijkstra (1994) further refines this by creating a fibrolytic bacteria pool and splitting amylolytic “microbes” into amylolytic bacteria and protozoa. In the Dijkstra models, inputs to a given microbial pool are represented as a microbial growth yield constant multiplied by the corresponding uptake of growth substrate (e.g., soluble protein or ammonia) for growth, with growth substrate requirement factors adapted from Reichl and Baldwin (1975). Additionally, these inputs apply specifically to the polysaccharide-free microbial dry matter, as amylolytic storage polysaccharides are represented as a separate state variable pool. While most kinetic parameters are shared between the amylolytic and fibrolytic groups, they differ in outflow rates and composition (Dijkstra et al., 1992), as only amylolytic bacteria synthesize storage polysaccharides. While amylolytic bacteria do not utilize this polysaccharide to power cellular processes, amylolytic hexose is taken up for formation of amylolytic bacterial storage polysaccharide. This may impact microbial growth, which depends on energy substrate availability, and distinguish the growth dynamics of each bacterial pool.

Mills et al. (2014) extends Dijkstra (1994) by splitting the amylolytic bacterial pool into lactate-producing bacteria and lactate-utilizing bacteria. Lactate-producers ferment amylolytic hexose to either lactate or VFA, depending on specific growth rate and rumen pH, while lactate-utilizers use both lactate and amylolytic hexose as energy sources. Lactate utilization by protozoa is also included. The growth of these microbial pools is represented similarly to that of Dijkstra (1994) and Dijkstra et al. (1992). Muñoz-Tamayo et al. (2016) uses a different approach from the prevailing method of disaggregation by carbohydrate utilization type, instead representing the rumen microbiota with three functional groups: sugar (glucose) utilizers, amino acids utilizers, and hydrogen utilizers. In Muñoz-Tamayo et al. (2016), ammonia is assumed to be the sole N source for sugar utilizers, and microbial growth is represented as a growth yield constant multiplied by the corresponding substrate uptake flux.

In Dijkstra et al. (1996b), uptakes of the aggregated microbial pool include outflow from the rumen, where the outflow rate for the aggregated microbial group is estimated by assuming that the total microbial biomass comprises 40% protozoa and 60% bacteria, that particle-associated bacteria make up 75% of the total bacterial biomass, and that the fractional outflow rate of protozoa is half of that of the fluid outflow rate. The need for such assumptions demonstrates the limitations of using aggregated microbial pools for more complex modeling exercises. This approach does not account for protozoal predation and resultant microbial N recycling in the rumen. In contrast, the 1992 and 1994 Dijkstra models with disaggregated amylolytic and fibrolytic microbe include outflows from each pool corresponding with the appropriate fraction, e.g., all amylolytic microbes flow out with the fluid and that all fibrolytic microbes with the solid fraction. In addition, microbes can be taken up via predation by protozoa and protozoa can also die due to lysis.

In Muñoz-Tamayo et al. (2016), the only uptakes of each functional microbial group are through death, represented as a mass-process where the death rate is constant across microbial groups. In France et al. (1982), uptake from the aggregated microbial group also includes microbial death, parameterized with a death rate dependent on the specific rate of microbial catabolism, which itself depends sigmoidally on the microbial growth rate, as well as washout to the omasum. Baldwin et al. (1977) includes microbial uptakes such as outflow of liquid-associated microbes with the rumen fluid, while particle-associated microbes pass out in association with insoluble particles. In Baldwin et al. (1987), it is assumed that large-particle associated microbes do not pass out of the rumen, while those in the small-particle-associated microbial pool (corresponding to the earlier model’s particle-associated pool) and fluid-associated pools do.

As rumen mechanistic models have evolved to predict CH_4_ production, the increasingly complex representations of fermentative pathways and other controls have not been matched by increasing complexity in representations of fermentative microbial groups. The only models currently incorporating thermodynamic control of fermentation pathways (van Lingen et al., 2019, 2021) represent all fermentative microbes by a single pool. Sensitivity analysis in van Lingen et al. (2019) identified the fiber degradation rate constant as influential on CH_4_ predictions, so distinguishing between cellulolytic and amylolytic bacteria may improve representation of substrate degradation, fermentative substrate availability, and CH_4_ production (van Lingen et al., 2019). Less aggregation of bacteria solely based on carbohydrate utilization and inclusion of additional bacterial functional groups in the rumen may also be necessary to improve CH_4_ predictions, especially under supplementation of CH_4_-inhibitors. AFA targeting methanogens may provide an advantage to reductive acetogens to compete for H_2_ (Ellis et al., 2008)_,_ but utilization of H_2_ by reductive bacteria is not included in any extant model of rumen fermentation. Similarly, sulfate-reducing bacteria, while minor under normal conditions, may become more prevalent H_2_-utilizers under CH_4_-inhibition or alternative feeding practices that include sulfur-containing feeds, such as corn co-products, but are not included in any extant rumen model (Ellis et al., 2008). Inclusion of additional microbial functional groups may be an important next step for more biologically realistic representation of H_2_ balance in the rumen. This endeavor can be supported by the increased knowledge of the diversity and function of the rumen microbiome generated through genomic approaches (Seshadri et al., 2018). However, this expanded genomic information has not yet been matched by representations of microbial metabolism in rumen mechanistic models (Bannink et al., 2016), although recent work has integrated microbial “omics” data into dynamic models of the metabolism of *Fibrobacter succinogenes* (Fakih et al., 2023) and the entire rumen microbiome (Davoudkhani et al., 2024). Further integration of omics data into rumen mechanistic models can improve representation of both microbial metabolism and fermentation stoichiometry (Muñoz-Tamayo et al., 2023) and mechanisms of CH_4_-inhibition by AFA (Indugu et al., 2024; Tan et al., 2024), potentially improving predictions of CH_4_ emissions.

#### 4.2.2 Protozoa and protozoal storage polysaccharides

The only extant rumen model that explicitly includes protozoa is Dijkstra (1994) and its derivatives. In Dijkstra (1994), inputs to the protozoa pool represent the uptake by protozoa of many substrates, and protozoal growth is determined by the minimum of either the growth supported by engulfed carbohydrates or protein. Inputs to the protozoal storage polysaccharide pool are yield constants multiplied by the corresponding uptakes of energy substrates. The representation of protozoal growth in Mills et al. (2014) is the same as in Dijkstra (1994), with the addition of lactate as an energy substrate for protozoal growth, and maximum protozoal uptake of bacterial protein and feed protein depends on rumen fluid pH.

To represent the high observed rate of protozoal lysis in the rumen in Dijkstra (1994), thought to be caused by unrestricted soluble sugar uptake and intracellular accumulation of acidic fermentative end products, protozoal lysis is included as an uptake, which is sigmoidally dependent on the rate of VFA production from hexose fermentation and protozoal biomass. Uptakes of protozoa also include outflow from the rumen, which, due to protozoal sequestration, is assumed to be 45% of the solid outflow rate. Thus, all protozoa are eligible for lysis, regardless of whether they are sequestered with less access to soluble substrates, leading to a rapid increase in protozoal death rates at high nutrient availabilities and mimicking the high protozoal lysis rates observed *in vitro*. However, recent work has questioned the applicability of these rates *in vivo*, due to limitations in studies that led to high observed lysis rates (Firkins et al., 2007). Diaz et al. (2014) postulated that extensive autolysis by isotrichids in culture tubes may result from their inability to migrate away from lytic conditions, unlike in the *in vivo* environment, where they could sequester in the ventral rumen. Therefore, the true *in vivo* protozoal lysis rate may be lower than that represented in Dijkstra (1994). Given the important role of protozoa in sequestering soluble substrates, revisiting the representation of protozoal lysis may improve predictions of VFA production and CH_4_ emissions.

#### 4.2.3 Anaerobic fungi

No mechanistic rumen model includes anaerobic fungi, perhaps because of their complex life cycle and relatively poorly characterized metabolism. However, France et al. (1990) uses a mechanistic, differential equation-based model to predict the proportion of the fungal population in each life stage (zoospore, immature thalli, and mature thalli) and total population size. Inclusion of anaerobic fungi in future models is important due to their impact on insoluble particle degradation (Kennedy and Murphy, 1988) and, consequently, particle retention and outflow rates. In addition, rumen anaerobic fungi play an important role in interspecies H_2_ transfer, and therefore, CH_4_ production. Methanogens are thought to be ecto-symbionts of anaerobic fungi, which, like protozoa, produce large amounts of H_2_ gas via specialized hydrogenosome organelles (López-García et al., 2022; Valle et al., 2015). Therefore, representing interspecies H_2_ transfer between anaerobic fungi and methanogens may improve predictions of CH_4_ production and H_2_ accumulation under CH_4_-inhibition. To advance representation of anaerobic fungi in future models, the approach of France et al. (1990) could potentially be incorporated into a full rumen model.

### 4.3 Fermentative products

The products of microbial carbohydrate fermentation (VFA, as well as other organic acids such as lactic acid) are the primary energy source for the ruminant host and different VFA species are relative sources or sinks of H_2_, impacting CH_4_ production. While almost all models consider the protonated (acid) and deprotonated (conjugate base) forms of the fermentative products together in one aggregated pool, this section will refer to all products in their acid form unless specifically referring to the disassociated form. Typical representations of fermentative products in models are reviewed below and an overview is presented in Figure 3B.

#### 4.3.1 Major VFA (acetic, propionic, and butyric acids)

The production of the major VFA (acetic, butyric, and propionic acids) is generally represented in the same manner mathematically, using empirically-derived stoichiometric yield factors to give relative proportions of each VFA species, based on high-forage or high-starch diets and usually interpolated for intermediate diets. This approach is more thoroughly examined in Alemu et al. (2011) and Bannink et al. (2006). Inputs to each VFA pool (acetic, butyric, propionic, and valeric acids) in Dijkstra (1994), include VFA content of the feed and fermentation of hexose, additional energy substrates available to protozoa, or soluble protein for growth, non-growth, and storage polysaccharide formation by microbes, according to stoichiometric yield factors. Dijkstra et al. (1996b), a simplified version of Dijkstra (1994) and Dijkstra et al. (1992), contains five inputs to an aggregated VFA pool: intake with diet, fermentation of soluble carbohydrates for microbial growth and for non-growth processes, and soluble protein fermentation. However, instead of predicting the VFA species, it uses an empirical average VFA profile for the diet simulated by the model.

Baldwin et al. (1977) represents VFA production using a relatively high degree of aggregation. Total VFA production is calculated using a fermentation balance equation, while acetic, propionic, butyric, and unspecified “higher” acids are given as proportions of total VFA using static stoichiometric relationships. Baldwin et al. (1987) disaggregates the major VFA and explicitly represents acetic, propionic, and butyric acid pools using empirical stoichiometric yield factors; Danfær et al. (2006) use a similar approach. Muñoz-Tamayo et al. (2016) uses a novel approach to modeling the production of the three major VFA, defining fermentation stoichiometry through biochemical reactions. This approach reduces the number of model parameters by avoiding unknown stoichiometric factors based on diet, arguably making it more mechanistic. van Lingen et al. (2019) uses a similar approach, utilizing biochemical reactions to predict individual VFA species formation.

In Dijkstra (1994) and Dijkstra et al. (1992), uptakes from each VFA pool include outflow from the rumen with the fluid fraction and absorption through the rumen wall. The latter is represented using modified Michaelis–Menten kinetics, where the maximum absorption rate is dependent on rumen wall surface area and is modified sigmoidally by rumen fluid pH to represent acid disassociation, with slower absorption rates of disassociated acids at higher pH. Dijkstra et al. (1996b) simplifies this representation by assuming a constant absorption rate based upon empirical VFA absorption data at rumen fluid pH values typical of sugarcane-based diets. Baldwin et al. (1987) represents the uptake of each major VFA with one aggregated mass-action reaction representing both outflow and absorption. Muñoz-Tamayo et al. (2016) includes an uptake of acid disassociation to the ionized conjugate base.

**Figure 4:**
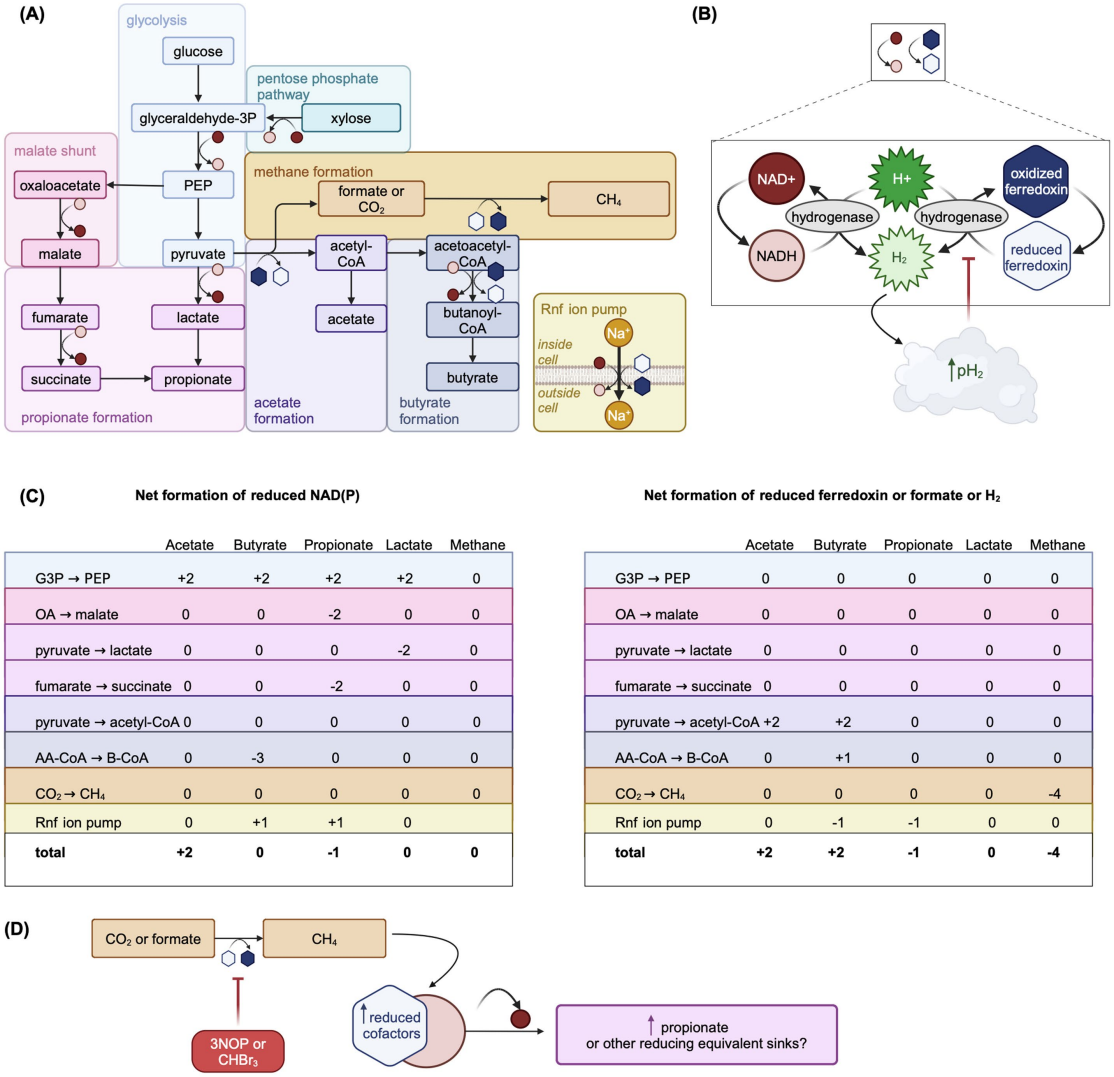
Overview of carbohydrate fermentation pathways in the rumen emphasizing the net production of reduced cofactors by each pathway and the re-oxidation of reduced cofactors. Adapted from Figures 1, 3, 4, and 6 in Hackmann, 2024, used under CC BY 4.0 (https://creativecommons.org/licenses/by/4.0/). **(A)** Summary of key fermentation pathways in the rumen with symbols representing the oxidation or reduction of cofactors in each reaction step. See **(B)** for key to redox symbols. Steps in each pathway may be combined or not shown for simplicity. **(B)** Key to redox symbols in Panel **(A)** and overview of hydrogenase-catalyzed cofactor re-oxidation. If H_2_ produced through cofactor re-oxidation accumulates, the rumen pH_2_ can increase which is thought to thermodynamically inhibit fermentation pathways that lead to net cofactor reduction. Hydrogenase represents a generic hydrogenase. Reduced cofactors can also be re-oxidized via bifurcating hydrogenases, which is not shown. Reoxidation reactions do not necessarily show balanced stoichiometries. **(C)** Summary of the net reduced cofactors NAD(P) (left sub-panel) and reduced ferredoxin (right sub-panel) generated in the steps of fermentation pathways shown in **(A)**. **(D)** Hypothetical scheme of fermentation pathway redirection to fermentation products that are net sinks of reduced cofactors, such as propionate, under inhibited methanogenesis. AA-CoA: acetoacetyl-CoA, B-CoA: butanoyl-CoA, CHBr_3_: bromoform, CoA: coenzyme A, G3P: glyceraldehyde 3-phosphate, OA: oxaloacetate, PEP: phosphoenolpyruvate. Created in BioRender. Pressman, E. (2024d) https://BioRender.com/i01j227.

More complex representations of VFA production have developed in concert with increased interest in representing CH_4_ production. It is likely that using empirical stoichiometric yield factors leads to errors in predicting relative VFA proportions and production, partly due to measurement errors in the data upon which the empirical factors are developed. Because direct observation of VFA production rates *in vivo* is technically difficult, it is typically assumed that molar proportions of VFA in rumen fluid are representative of the proportions in which VFA are produced (Alemu et al., 2011). However, this assumption is not necessarily valid given differential rates of VFA absorption and utilization in the rumen epithelium or by the rumen microbiota. Such stoichiometric constants also assume a fixed fermentative pattern for all microbial subpopulations, even though fungi and protozoa produce very little propionate, and a fixed microbial growth efficiency with substrate fermentation to each VFA. Finally, VFA interconversion cannot be represented using these fixed factors, nor do they incorporate the effect of pH on rumen VFA stoichiometric coefficients, potentially affected through interplay with thermodynamics and hydrogenase-mediated conversion of H+ to H_2_ (see Section 4.5 and Figure 4). Only recently have models of Muñoz-Tamayo et al. (2016) and van Lingen et al. (2019) used yield factors from balanced fermentation reaction equations for hexose and protein fermentation. This type of representation requires its own assumptions, e.g., that non-hexose monosaccharides have the same fermentation stoichiometry as hexose or the structure of the “average” amino acid.

#### 4.3.2 Lactic acid

Mills et al. (2014) modifies Dijkstra (1994) to include lactic acid metabolism in the rumen (Figure 3B). The production of lactic acid is similar to that of the major VFA in Dijkstra (1994). Inputs to the lactic acid pool include intake with the diet and fermentation of hexose by lactate-producing amylolytic bacteria. The proportion of hexose fermented to lactic acid is assumed to increase as specific growth rate increases and as pH decreases. Uptakes of lactic acid in Mills et al. (2014) include outflow with fluid, absorption through rumen wall, and fermentation to VFA by lactate-utilizing bacteria and protozoa; uptakes for fermentation to VFA are all inputs to the corresponding VFA pool.

While the balance of lactic acid production and utilization usually prevents its accumulation in rumen, at high microbial growth rates and increased glycolytic flux, the production of more reduced products like lactic acid is a means to rapidly regenerate NAD+, whereas production of more oxidized products like acetic acid would be inhibited by the high H_2_ partial pressure (Mackie et al., 1984) (Figures 4A,C). Therefore, the inclusion of lactic acid metabolism may gain importance as thermodynamic control of fermentative pathways becomes more prominent in rumen models. In addition, including lactic acid metabolism allows for more mechanistic prediction of rumen fluid pH.

#### 4.3.3 Minor VFA and other fermentative end-products

In addition to the major VFA and lactic acid, some models also include minor VFA species. For example, Dijkstra (1994) and Dijkstra et al. (1992) incorporate hexose fermentation to valeric acid. However, no current mechanistic models of rumen fermentation include minor VFA (such as caproate), formate, or organic acids like fumaric or malic acid, despite their potential importance as intermediates in propionate or CH_4_ production (Ellis et al., 2008). Only Danfær et al. (2006) includes an aggregated branched-chain VFA pool, produced through protein fermentation. While branched-chain VFA are minor species, they are important intermediates in branched-chain amino acid catabolism and microbial growth, suggesting that their inclusion could improve the representation of microbial growth dynamics and protein metabolism in the rumen. Additionally, incorporating alternative fermentative substrates and products, such as formate, malate, and fumarate, may improve predictions of fermentative shifts under CH_4_-inhibition (Ellis et al., 2008).

### 4.4 Rumen pH

Few models include mechanistic control of rumen pH and misrepresentation of pH is a major contributor to errors in CH_4_ prediction (Bannink et al., 2011). Approaches to modeling dynamic rumen pH include charge balancing and kinetic modeling of organic disassociation with bicarbonate buffering. Dijkstra (1994) and Dijkstra et al. (1992) use a static rumen fluid pH while Baldwin (1995) employs an empirical approach based on relative proportions of VFA and lactate without accounting for saliva’s buffering effect. The model of Muñoz-Tamayo et al. (2016) mechanistically represents dynamic rumen pH by explicitly modeling acid–base pair disassociation and solving for H+ concentration. Imamidoost and Cant (2005) use a more typical rate: state formula to depict rumen pH where the dissociation of an aggregated organic acid state variable is controlled by bicarbonate buffer content, and dynamic rumen pH is then calculated using the Henderson-Hasselbach equation. Offner and Sauvant (2006) also use the rate: state formalism to explicitly represent hydrogen ions as a state variable with flows from VFA and bicarbonate disassociation, and through H_2_ pool via redox reactions.

Rumen pH can affect fractional VFA absorption rates (Alemu et al., 2011) as well as fiber degradation by rumen microbes (Dijkstra et al., 1992). One study found that errors in rumen acidity had the greatest effect on estimated CH_4_ emissions, where a 0.1 reduction in rumen pH decreased estimated CH_4_ emission by over 3% (Bannink et al., 2011). Similarly, differences in fractional absorption rates among VFA at lower rumen pH could partly explain variations in model predictions (Dijkstra et al., 1993). Given its important role in VFA profile and interactions with redox reactions, further development of mechanistic models for dynamic rumen fluid pH is warranted.

### 4.5 Redox balance in the rumen

Mechanistic representation of redox reactions and thermodynamic control in rumen models is relatively limited, but increasingly recognized as important (van Lingen et al., 2016, 2019). Below, aspects of rumen redox balance, including CH_4_ and methanogens, are discussed.

#### 4.5.1 Metabolic hydrogen and other electron donors

Early representations of H_2_ production in the rumen treated H_2_ as a “zero pool^”^ (France et al., 1992), whereby the difference between all explicitly represented H_2_ inputs and outputs was used for CH_4_ production as seen in Baldwin (1995), Benchaar et al. (1998) and Mills et al. (2001). Similarly, Danfær et al. (2006) models CH_4_ production based on empirically based on stoichiometric fermentation coefficients without representing methanogens. Mills et al. (2001) modified the Baldwin (1995) model to include H_2_ production from the fermentation of carbohydrate and protein substrates to acetate and butyrate and microbial growth on amino acids. H_2_ uptake was represented for microbial growth on NPN, biohydrogenation of unsaturated fatty acids and fermentation of substrates to propionate and valerate. Vetharaniam et al. (2015) also adapted Baldwin (1995) to simulate the rumen of a sheep and predict CH_4_ emissions, using the same H_2_-balance approach as Mills et al. (2001).

More recent models (e.g., van Lingen et al., 2019) instead represent an H_2_ pool using the rate: state formalism. Inflows include carbohydrate fermentation to acetate and butyrate, with absorption and outflow of dissolved H_2_ explicitly represented using Henry’s law and the ideal gas law. Eructation of gaseous H_2_ is represented using a mass-action process and H_2_ uptake for methanogen growth is modeled using Michaelis–Menten kinetics. While van Lingen et al. (2019) does not represent protein metabolism or H_2_ production through amino acid fermentation, this flux is included in Muñoz-Tamayo et al. (2016) via mass-action equations. Muñoz-Tamayo et al. (2016) also explicitly represents non-equilibrium liquid–gas transfer of H_2_ from dissolved to gaseous state using mass-action kinetics with dissolved H_2_ uptake for methanogenesis and microbial growth on NPN, both represented by Michaelis–Menten kinetics.

No rumen mechanistic model currently represents methanogenic electron donors or substrates besides H_2_ and CO_2_. Formate, produced by anaerobic fungi and protozoa, may contribute up to 18% of CH_4_ production (Hungate et al., 1970) and serve as an electron donor for reducing fumarate and malate to succinate and then propionate. Including formate and other potential electron donors like acetate, methanol, and methylamines in models could improve predictions of CH_4_ emissions and VFA profiles, especially under CH_4_-inhibition (Ungerfeld, 2020).

#### 4.5.2 Microbial cofactors and thermodynamic control of fermentation pathways

Accurate CH_4_ prediction depends on correctly modeling relative concentrations of VFA produced through fermentation (Alemu et al., 2011), which typically first proceeds via glycolysis (Figure 4A). NADH and reduced ferredoxin, cofactors carrying electrons, must be re-oxidized for glycolysis to proceed (Hegarty and Gerdes, 1999) (Figure 4B). Although acetate is the most abundant VFA, its production from pyruvate is not directly coupled to the re-oxidation of reduced cofactors, unlike propionate (Figure 4C). When acetate is produced from pyruvate, cofactors must be re-oxidized via hydrogenase-catalyzed oxidation, which results in production of H_2_. This is thermodynamically inhibited when the rumen hydrogen partial pressure (pH_2_) is elevated (van Lingen et al., 2016) (Figure 4B). Thus, pH_2_ thermodynamically controls fermentation pathways via the ratio of oxidized to reduced cofactors (van Lingen et al., 2016), impacting VFA molar ratios, H_2_ balance, and ultimately CH_4_ production. Thermodynamic favorability of fermentation pathways may also interact with other rumen conditions, such as pH, as hydrogen ions are taken up for hydrogenase-catalyzed NADH oxidation to NAD+ (van Lingen et al., 2016). Incorporation of thermodynamically controlled cofactor dynamics improved the prediction of VFA under different pH, glucose concentration, and pH_2_ conditions in a model of *in vitro* mixed culture fermentation (Zhang et al., 2013). Only two models, van Lingen et al. (2019, 2021) and Offner and Sauvant (2006) represent microbial fermentative cofactors and their thermodynamic control on fermentation pathways mechanistically.

van Lingen et al. (2019, 2021) explicitly represents the NAD+/NADH ratio, controlling flux allocation between carbohydrate fermentation pathways. Inflows to the oxidized NADH pool include hexose fermentation to acetate, while outflows include hexose fermentation to propionate and hydrogenase-catalyzed reoxidation. Elevated pH_2_ inhibits NADH reoxidation by modifying the mass-action reoxidation process via the thermodynamic potential factor (Jin and Bethke, 2007). Offner and Sauvant (2006) use a more complex approach, predicting thermodynamically favorable fermentation pathways by explicitly modeling Gibbs free energy changes. Muñoz-Tamayo et al. (2021) uses a hybrid approach assuming a linear relationship between NADH/NAD+ and pH_2_, with pH_2_ controlling flux allocation parameters. More widespread adoption of thermodynamic control in rumen models could further improve CH_4_ predictions.

#### 4.5.3 Methanogens and CH_4_

Only van Lingen et al. (2019, 2021) explicitly include methanogens and methane using the rate: state formalism (Figure 3A), while Muñoz-Tamayo et al. (2016, 2021) include a “hydrogen-utilizers” microbial pool, corresponding to methanogenic archaea. In van Lingen et al. (2019), methanogen growth via hydrogenotrophic methanogenesis is modeled by a growth yield constant multiplied by H_2_ uptake. Methanogen outflow is set to 40% of the liquid and solid outflow rates, reflecting the slower outflow of methanogens due to their adherence to rumen epithelium. The CH_4_ production rate is then modeled by multiplying the H_2_ uptake flux for methanogen growth by a yield rate. In Muñoz-Tamayo et al. (2016), dissolved CH_4_ production is represented as a yield factor multiplied by the H_2_ uptake for H_2_-utilizer growth, and conversion to gaseous CH_4_ occurs via liquid–gas transfer.

Future models could benefit from more detailed representation of methanogenic subpopulations. Methanogen species vary in several respects that are relevant to predicting the impact of AFA, such as sensitivity to CH_4_-inhibitors, H_2_ affinity, and methanogenic pathways. *Methanobrevibacter ruminantium* is particularly sensitive to halogenated sulfonated methyl–coenzyme M reductase (MCR) inhibitors (Patra et al., 2017), potentially due to this species’ inability to synthesize coenzyme M (Ungerfeld and Pitta, 2024). Halogenated CH_4_ analogs such as bromoform reduce CH_4_ production by inhibiting cobamide-dependent methyl transfer (Wood et al., 1968). It is speculated that differences in methanogen sensitivity to halogenated CH_4_ analogs may also be due to differences in cobamide structures and affinities for these analogs, but this has yet to be investigated experimentally (Ungerfeld and Pitta, 2024). Differences in expression of MCR isozymes may also contribute to variability in sensitivity to AFA, as well as H_2_ affinity, across methanogen species (Pitta et al., 2022b; Ungerfeld and Pitta, 2024).

Methanogens also differ in methanogenic pathways, although extant mechanistic rumen models incorporate only hydrogenotrophic methanogenesis. Methylotrophic methanogenesis requires only one mole of H_2_ to produce CH_4_ from methyl group-bearing substrates, while hydrogenotrophic methanogenesis requires three or four moles of H_2_ (Ungerfeld and Pitta, 2024). Thus, increased abundance of methylotrophic methanogens could contribute to greater CH_4_ yields, but is limited by the availability of methyl group-bearing substrates (Feldewert et al., 2020). On the other hand, the lower H_2_ threshold of methylotrophic methanogens may be less of a competitive advantage under CH_4_-inhibition, when pH_2_ in the rumen is higher. As pectin and xylan are rich in methyl groups (Feldewert et al., 2020), basal diet also may partially determine methanogen population structure and the impact of AFA on methanogenesis. However, the representation of non-hexose soluble carbohydrates is simplified in rumen models. Baldwin et al. (1987) specifies pectin as its own input, but simulates its fermentation using a combined soluble carbohydrates pool, and the *α*-hexose group in France et al. (1982) includes pectin. This simplified representation of complex plant polysaccharides and methanogen community structure could impact CH_4_ prediction accuracy by underestimating methylotrophic methanogenesis under normal conditions and overestimating it under CH_4_-inhibition.

Methanogenic microbiomes are likely influenced by many interacting factors such as basal diet, host genetics (Pitta et al., 2022b), and bacterial community composition, which has complex dynamics itself under CH_4_-inhibition (Ungerfeld and Pitta, 2024). Mechanistic models may be an ideal tool for predicting changes in methanogen community structure under CH_4_-inhibition. However, mechanistically modeling methanogens with more complexity would require model inputs (such as initial conditions of methanogen population distributions) and parameters (such as affinity constants and growth yields) that are difficult to obtain or may yet be unknown, making modeling methanogenic subpopulations under typical or CH_4_-inhibited conditions challenging.

#### 4.5.4 CH_4_-inhibiting feed additives

Only two mechanistic models incorporate AFA: van Lingen et al. (2021) for 3-nitroxypropanol (3NOP) and Muñoz-Tamayo et al. (2021) for bromoform from *Asparagopsis taxiformis*. While these models assume that AFA only act directly on methanogens, it is possible but currently unknown if AFA directly affect some rumen bacteria as well (Ungerfeld and Pitta, 2024). In addition to directly inhibiting MCR, 3NOP may redirect fermentation pathways toward propionate formation (van Gastelen et al., 2022), possibly through thermodynamic inhibition of NADH re-oxidation (van Lingen et al., 2021). A potential scheme of this redirection is shown in Figure 4D. These interactions emphasize the need for models to include both AFA and microbial cofactor dynamics.

The potential loss of persistent CH_4_-inhibition by 3NOP in some studies (Schilde et al., 2021; Van Gastelen et al., 2024) suggests that microbial adaptation to AFA may be possible. However, such adaptation is not included in any rumen mechanistic model, and mechanistic models are often evaluated assuming quasi-steady state conditions (Danfær et al., 2006; Dijkstra, 1994; van Lingen et al., 2019, 2021). However, the time-dependent nature of microbial adaption aligns with suggestions by Muñoz-Tamayo et al. (2019) that models using substrate utilization kinetics alone cannot accurately predict methanogen dynamics, underscoring the importance of incorporating spatial and temporal information in models of H_2_ utilization. For example, 3NOP supplementation may reduce MCR expression (Pitta et al., 2022a), so methanogenic adaptation to AFA may also be related to upregulation of MCR expression or expression of alternate MCR isoenzymes or subunits. Explicit modeling of fluctuations in MCR expression over time, potentially incorporating genomic data (e.g., Pitta et al., 2022a), may allow for predictions of methanogen adaptation to AFA.

AFA may also have indirect effects on net GHG reductions. For example, 3NOP is metabolized to NO_3_− and NO_2_−in the rumen (Duin et al., 2016), which can further be reduced to nitric oxide (NO) and ultimately nitrous oxide (N_2_O). While enteric N_2_O emissions are typically minor, denitrifying genes are present within the rumen bacterial and archaeal metagenome (Latham et al., 2016) and 3NOP supplementation may increase enteric N_2_O emissions (Petersen et al., 2015). In addition, application with manure from cows fed 3NOP led some soils to emit more N_2_O (Weber et al., 2021), suggesting potential alterations to N-excretion. As N_2_O is a potent GHG with a greater global warming potential than CH_4_ (IPCC, 2023), models of enteric fermentation under CH_4_-inhibition should consider the potential indirect and downstream impacts on GHG production.

Approaches that assume the anti-methanogenic action of *Asparagopsis* species is due to solely its bromoform content allow for standardization of the inhibitory effect of various macroalgae species but may overlook compounds that act synergistically with bromoform (Ahmed and Nishida, 2024; Machado et al., 2016). Because different AFA have different mechanisms of CH_4_-inhibition, it is possible they can also act synergistically (Villar et al., 2020), although this effect has not been seen consistently *in vivo* (Guyader et al., 2015; Maigaard et al., 2023; Zhang et al., 2021). Currently, no rumen mechanistic model represents direct supplementation of multiple AFA of different mechanisms, although van Lingen et al. (2021) does includes 3NOP catabolism to NO_3_− and NO_2_−.

### 4.6 Rumen fractional outflow rates

Mechanistic incorporation of rumen passage rates in fermentation models is rare. Models by Dijkstra (1994) and Dijkstra et al. (1992) assume constant outflow rates for fluid and solid fractions. Danfær et al. (2006) uses a more flexible approach, where passage rates for feed fractions are based on the passage rate of forage indigestible NDF, which itself depends on the ratio of NDF intake to animal body weight. France et al. (1982) mechanistically models rumen outflow based on rumen fluid pool size, where outflow to omasum is a flux from the fluid volume pool but uses one outflow rate for both soluble and insoluble digesta.

In the Baldwin lineage of models, rumen outflow is represented using particle size distribution-dependent solid outflow rates, where particles above a threshold size cannot leave rumen (Baldwin et al., 1987; Gregorini et al., 2015). Inflows to each particle size pool are based on the solubility of dietary nutrients. Large particles flow into the small particle pool via particle size reduction due to rumination. Outflows include liquid-associated small particles, and size comminution by hydrolysis by associated microbes. Gregorini et al. (2015) updated MOLLY to include three particle size pools, and water absorption through the rumen wall due to increased rumen fluid osmolality. The rumen fluid pool size determines the rate of liquid passage according to a mass-action process. Some specialized models focusing only on particle and fluid dynamics in the rumen predict fluid outflow based on more complex factors such as reticulo-omasal orifice activity (Seo et al., 2007) or particle movement through the rumen in a two-pool model (Poppi et al., 2001). However, these models have not been integrated with more comprehensive models of rumen fermentation.

Rumen fermentation patterns depend on digesta outflow rates, and overly simplistic representations can lead to errors in predicted CH_4_ production (van Lingen et al., 2019). Rumen outflow is influenced by feed intake, composition, feed particle size, and microbial fermentation rate (Gregorini et al., 2015). Sensitivity analysis in previous rumen models have implicated rumen passage rates as highly influential on modeled CH_4_ production (Bannink and De Visser, 1997; Neal et al., 1992). In van Lingen et al. (2019), parameters determining rumen fractional passage rates and NADH oxidation rate together explained 86% of the variation in predicted daily CH_4_ emission. Precise estimation of rumen outflow rates may be particularly important when modeling fermentation under CH_4_-inhibition, as outflow rates may interact with AFA’s inhibitory effect. For example, 3NOP is water-soluble and its retention in the rumen and longevity of its inhibitory effect may depend on the liquid outflow rate (Reynolds et al., 2014; Vyas et al., 2018). More mechanistic representation of rumen fractional outflow rates may improve CH_4_ production predictions, especially under inhibitory conditions.

## 5 Discussion

Inhibiting methanogenesis in the rumen is of interest because CH_4_ is both a major GHG and a loss of potentially utilizable energy from ruminants. Because CH_4_ is considered a loss of GE, its inhibition is expected to return GE to the animal and improve feed efficiency. However, CH_4_-inhibition in ruminants does not consistently achieve production benefits (Morgavi et al., 2023). H_2_ emissions typically increase under CH_4_-inhibition, but energy losses through H_2_ are variable and generally not directly proportional to the energy “saved” by reduced CH_4_ emissions (Morgavi et al., 2023). While inhibiting methanogenesis is expected to lead to H_2_ accumulation, thermodynamic inhibition of NADH or reduced ferredoxin re-oxidation, and disruptions to fermentation, this effect has also not been observed consistently *in vivo* (Morgavi et al., 2023). However, variations in plasma (Yanibada et al., 2020) and milk (Yanibada et al., 2021) metabolites in dairy cattle suggest that alterations in energy partitioning do occur under CH_4_-inhibition. These changes could potentially occur concomitantly with H_2_ redistribution through increased microbial protein synthesis, as microbial protein may act as an electron sink (Morgavi et al., 2023), or changes in nutrient metabolism, such as increased production of propionate, a glucogenic precursor and H_2_ sink. A better understanding of the metabolic fate of excess H_2_ under inhibited methanogenesis and the potentially utilizable “saved” GE requires more detailed representation of the thermodynamic favorability of fermentation pathways and enhanced descriptions of rumen microbial activities, alternative fermentation pathways, and H_2_ sinks (Ellis et al., 2008; Morgavi et al., 2023).

As reviewed herein, the interplay of the thermodynamic favorability of fermentation pathways, microbial activities, and diet could be further developed to predict how excess H_2_ is apportioned to reductive acetogenesis, sulfate-or nitrate-reduction, biohydrogenation, propionate formation, microbial growth, or additional pathways. The models of van Lingen et al. (2021) and Muñoz-Tamayo et al. (2021) are the first to represent rumen fermentation with detailed representation of metabolic pathways yielding H_2_, CH_4_, and different VFA, as well as CH_4_ -inhibiting AFA. However, both Muñoz-Tamayo et al. (2021) and van Lingen et al. (2021) lack representation of biohydrogenation and the former does not include biomass growth on ammonia, omitting potentially important H_2_ sinks under CH_4_-inhibition. Additional H_2_ uptakes include redirection to propionate (Section 4.5.4) or utilization by other microbial groups. As discussed in Section 4.2.1, increased representation of competition for H_2_ by reductive acetogenic or sulfate-reducing bacteria (Ellis et al., 2008) may improve predictions of CH_4_ production and H_2_ dynamics under certain dietary conditions. As these H_2_ sinks vary in their stoichiometric requirements for H_2_ (e.g., Reichl and Baldwin, 1975) and kinetic parameters (e.g., Ungerfeld, 2020), increasingly thorough representations of these pathways could improve predictions of CH_4_ production by more completely accounting for the dynamics of H_2_ uptakes and therefore H_2_ that remains as a methanogenic substrate.

While inclusion of these elements may improve mechanistic models as research tools to explore predicted changes in H_2_ and energy partition under CH_4_-inhibition, they can also be used to develop and test hypotheses for minimizing CH_4_ emissions from cattle. AFA efficacy depends on basal diet (Dijkstra et al., 2018; Kebreab et al., 2023), and time and amount of feeding have also been shown to impact methanogenesis, with more frequent and larger meals associated with lower CH_4_ yield in growing heifers (Biswas et al., 2022) and steers (Llonch et al., 2018). This effect may be due to alterations to the VFA profile, which may themselves be related to changes in rumen outflow rate and thus the rate and extent of organic matter fermentation (Crompton et al., 2011). van Lingen et al. (2021) is the only rumen model incorporating AFA and a diurnal feed intake pattern and therefore capable of evaluating the combined effects of feeding frequency and diet composition and degradation characteristics on rumen fermentation dynamics. However, this model includes static rumen outflow rates and cannot account for the potential effects of feeding level and frequency on rumen outflow. A model incorporating state variables and controls reviewed herein, such as AFA, complex representations of diet fractions, feed intake patterns, and rumen outflow, could be used to design basal diet and supplementation schedules that minimize CH_4_ emissions and test these *in vivo*.

However, data needs for the inclusion of these controls exemplify the trade-offs inherent to mechanistic modeling, whereby accurate predictions are typically only achievable given extensive inputs which may be practically infeasible to obtain (Ross et al., 2024). Increased synergies between *in vivo* AFA efficacy studies and modeling exercises, such as using optimal experimental design for model parameterization (e.g., Bandara et al., 2009; Morgavi et al., 2023) or using mechanistic models to design AFA optimization schemes to test *in vivo*, may help fill these data gaps. Some models are available online or are available in open-access software such as R (Beukes et al., 2020; Muñoz-Tamayo et al., 2021; van Lingen et al., 2021), but increased adoption of Open Science practices may accelerate the exchange of data and concepts and the improvement of rumen models (Muñoz-Tamayo et al., 2022). Simple yet accurate statistical approaches to predicting CH_4_ production under AFA supplementation, such as meta-analysis accounting for key explanatory variables (Dijkstra et al., 2018; Kebreab et al., 2023), may be useful tools as more studies in cattle under AFA supplementation become available. However, Kebreab et al. (2023) argue that the unknown mechanism of increased efficacy of 3NOP with greater starch and lower NDF content of the diet emphasizes the need for more complex mechanistic models to explain these relationships. Expansion of models of *in vitro* fermentation (e.g., Muñoz-Tamayo et al., 2016, 2021; Wang et al., 2013) to include additional H_2_ sinks such as lipid metabolism may be a compromise between statistical models and much more complex rumen models that still allows mechanistic interrogation of H_2_ allocation, including under CH4-inhibiting.

We here have comprehensively reviewed mechanistic models that make significant contributions to the mathematical representation of rumen fermentation. While some previous reviews have compared certain aspects of these models (Bannink et al., 2016; Bannink and De Visser, 1997; Ellis et al., 2008; Kebreab et al., 2009), none of these articles review more recent models that incorporate CH_4_-inhibiting AFA, nor do they discuss microbial and nutritional elements that are key to modeling the rumen under CH_4_-inhibition. We emphasized elements that should be included in future mechanistic models of rumen fermentation specifically under CH_4_-inhibition to more accurately predict microbial nutrient metabolism, H_2_ and energy partition, and CH_4_ emissions and thereby improve the use of mechanistic models as research tools. Currently, no rumen mechanistic model incorporates multiple AFA, thermodynamic control of VFA pathways, additional H_2_-utilizing microbial groups, and mechanistic control of rumen outflow and pH, although these controls may be critical for accurately predicting rumen fermentative dynamics under CH_4_-inhibition.
